# Research Ethics Boards: No Data on Quality of For-Profit or Non-Profit IRBs

**DOI:** 10.1371/journal.pmed.0030459

**Published:** 2006-10-31

**Authors:** Adil E Shamoo, Elizabeth Woeckner

We are concerned by two inaccurate statements offered by Emanuel [1]. First, he claims: “And in recent years, the OHRP [Office for Human Research Protections] has called upon WIRB [Western Institutional Review Board] to re-review protocols and revamp the IRB processes and procedures at not-for-profit academic institutions where the OHRP had temporarily suspended research.” We were sufficiently surprised by this that we sought clarification from OHRP Director Bernard Schwetz, who was kind enough to explain that Emanuel's statement was incorrect, and further, that “The Office for Human Research Protections has never asked WIRB to re-review protocols for any institution, nor would OHRP endorse any IRB in such a manner. OHRP will provide information about the several such IRBs. However OHRP does not suggest that we endorse any of these commercial or independent IRB services” [B Schwetz, personal communication].

Accordingly it is incorrect to conclude, as Emanuel does, that “Calling upon WIRB constitutes a vote of confidence by federal regulators that at least this one for-profit entity provides high-quality IRB review.”

Second, Emanuel writes: “OHRP has never suspended a for-profit IRB.” This is misleading and incorrect in that OHRP regulates institutions, not IRBs. OHRP Director Bernard Schwetz was kind enough to clarify that “While this statement is true, it is also true that OHRP has never suspended a not-for-profit IRB.”

It is useful to reflect upon the fact that WIRB has been the IRB of record in a number of egregious cases. For example: Faruk S. Abuzzahab, MD (medical license suspended following the death of research subject inter alia); Robert A. Fiddes, MD (indicted and convicted for research fraud); and Richard L. Borison, MD and Bruce I. Diamond, PhD (indicted and convicted for research fraud) [2]. Moreover WIRB was copied on the OHRP determination letters to Oregon Health and Science University regarding the Student Athlete Drug Surveillance Trial trial, the reasonable conclusion from which is that WIRB was involved in its review and approval [3,4].

There is no data, scientific or otherwise, because the lack of universal, legally codified human research protections discourages, if not prevents, collection of such information. We agree that commercial IRBs are not unacceptable simply because they review proposed research for a fee, but commercial IRBs may provide unacceptable review and oversight when they exist primarily to provide rapid approvals.

Finally, we would like to offer bibliographical addenda. Emanuel writes: “With Wood and Grady, I have proposed a system of regional ethics organizations that would review and monitor research protocols.” A recent paper reviewed the history of the proposals for regional IRBs [5]. The earliest suggestion for regional ethics organizations identified was Alberti (1995) [6].
